# Pictorial Personality Traits Questionnaire for Children (PPTQ-C)—A New Measure of Children's Personality Traits

**DOI:** 10.3389/fpsyg.2016.00498

**Published:** 2016-04-14

**Authors:** Marta Maćkiewicz, Jan Cieciuch

**Affiliations:** Institute of Psychology, University of Cardinal Stefan Wyszynski in WarsawWarsaw, Poland

**Keywords:** personality trait, Big Five, Exploratory Structural Equation Model, childhood, PPTQ-C

## Abstract

In order to adjust personality measurements to children's developmental level, we constructed the Pictorial Personality Traits Questionnaire for Children (PPTQ-C). To validate the measure, we conducted a study with a total group of 1028 children aged between 7 and 13 years old. Structural validity was established through Exploratory Structural Equation Model (ESEM). Criterion validity was confirmed with a multitrait-multimethod analysis for which we introduced the children's self-assessment scores from the Big Five Questionnaire for Children. Despite some problems with reliability, one can conclude that the PPTQ-C can be a valid instrument for measuring personality traits, particularly in a group of young children (aged ~7–10 years).

## Introduction

The Big Five model is a widely accepted model of personality description in terms of traits (De Raad and Perugini, 2002; McCrae and Costa, 2003; Matthews et al., 2009). McCrae and Costa (1997), the authors of the dominant Big Five variant in the literature, argue that the universal personality structure consists of five essential traits: extraversion, agreeableness, conscientiousness, neuroticism, and openness to experience. Given the simplicity of the description and the vast range of empirical evidence, McCrae (2009) compares the Big Five model to a universal physics of personality. In support of his strong proposition, McCrae (2009) quoted a number of studies, including cross-cultural and longitudinal studies, that demonstrated versatility and stability (McCrae, 2001; Hendriks et al., 2003; McCrae and Costa, 2003; McCrae et al., 2005). Although, there is some criticism of the Big Five model (Eysenck, 1992; Boyle, 2008; Cieciuch, 2012), its position in the literature seems to be quite strong.

Traditional research on personality traits based on the Big Five model (McCrae and Costa, 1997) has related mainly to adulthood. However, in recent years, there has been a significant increase in testing personality structure at earlier stages of development from childhood to adolescence (review of research in Shiner and Caspi, 2003; Caspi et al., 2005). The results of these studies allow us to theorize that personality structure among children and adolescents is highly similar to adult personality in terms of the Big Five (De Fruyt et al., 2000, 2009). However, there are also some typical differences in the personality factorial structure of children's and adults' personality structure; e.g., factor loadings are usually smaller, factor correlations are typically higher, or factors are less differentiated (Soto et al., 2008; Tackett et al., 2012; Morizot, 2014).

Studies on children's and adolescent's personality have taken three main approaches. Nevertheless, none is free of limitations. The first approach has used questionnaires that were designed for adults such as the NEO-FFI (Parker and Stumpf, 1998) and the NEO-PI-R (De Fruyt et al., 2000, 2009). is The results of the extensive cross-cultural research using the NEO-PI-R have shown that the personality structure of adolescents aged between 12 and 17 years is similar to the structure of the adult personality. The weakness of this approach the use of questionnaires intended for adults. For example, studies by De Fruyt et al. (2000, 2009) used the NEO-PI-R, which consists of 240 statements. These statements relate to various aspects of the life of an adult, which children would likely have not experienced (in this study, the youngest respondents were 12 years old).

The second approach has collected data obtained only from observers such as parents, teachers and peers. For example, Digman and Inouye (1986) showed that teachers' descriptions of children adopt a very similar structure to descriptions of adults based on the Big Five. Moreover, the results of the research of Mervielde et al. (1995) revealed that teachers' descriptions of children's personalities (aged 4–12 years old) followed the five-factor structure. Mervielde and De Fruyt (2000) also analyzed the personality descriptions made by peers. In the course of the analysis, not all factors were distinctly extracted. The analysis exhibited that descriptions of children's personalities are organized around three powerful factors: the first factor is a combination of intellect and conscientiousness, the second factor is extraversion combined with emotional stability, and the third factor is helpful behavior. Thus, the obtained results indicate a lower diversity of personality traits in children compared with adults. The weak point of this dominant approach is the use of data obtained only from observers (especially from parents and teachers). Descriptions of children's personalities by adult observers are the result of their observations of the children's personalities and their own cognitive categories and personalities. Furthermore, we should consider the fact that parents and teachers do not always have the opportunity to observe children in different situations.

In the third research approach, psychologists have attempted to construct self-report questionnaires that are suitable for children and adolescents. The representatives of this approach argue that data about children derived from other people's (teachers, parents) descriptions concern not the children themselves but rather the adults. One of the proposals is a questionnaire designed by Mervielde and De Fruyt (1999): the Hierarchical Personality Inventory for Children (HIPiC). The basis for the creation of this instrument was descriptions of children's personalities collected by research teams in seven countries (Kohnstamm et al., 1995). The data were organized into five factors: extroversion, friendliness (the equivalent of agreeableness), conscientiousness, emotional stability (the inverse of neuroticism), and imagination (the equivalent of openness to experience). The analysis of the self-descriptive data obtained from a group of adolescents indicated a high degree of convergent and discriminant validity with the HiPIC compared with the NEO-PI-R (De Fruyt et al., 2000).

In addition, Barbaranelli et al. (2003) developed a questionnaire to measure the Big Five dimensions in children and adolescents: the Big Five Questionnaire for Children (BFQ-C). It consists of 65 statements to which children must respond using the scale. Each of the five factors (extraversion, agreeableness, conscientiousness, emotional instability, and openness) consists of 13 test items. The criteria validity results for the BFQ-C showed a positive relationship between openness to experience, conscientiousness, and school achievement (Barbaranelli et al., 2003). The weakness of this approach is that it was mainly a traditional, verbal-based questionnaire. However, it consisted of short and simple statements and fewer items than the adult version of the scale.

### Children as a research group—characteristics, limitations, and challenges

A number of difficulties associated with the implementation of this plan are primarily associated with the incompatibility of questionnaire methods for measuring the cognitive development levels of the respondents. An important question is what type of measurement would be most appropriate for examining the structure of personality in children. Caspi et al. (2005) postulated that studies on the structure and development of children's personalities must go beyond the traditional instruments; the methods used in studies on children's personalities should be well suited to their development levels.

However, the period that is commonly referred to as middle childhood (6–12 years; Harter, 1999) lacks a reliable and valid measure of personality. In Piaget's theory (1960), middle childhood is a time when children function at a concrete level. They are focused on concrete, non-abstract elements of the world. They organize and generalize information about phenomena, things and people. At the same time children have developed a basic understanding of who they are (Harter, 1996; Eder and Mangelsdorf, 1997; Thompson et al., 2006), and they are able to compare themselves and others at different points in time, and they have a notion of stability. Thus, children in middle childhood can think about themselves as people who are characterized by a stable set of traits. They can specify which categories best suit them and reliably describe their behavior.

The main feature of the concrete operational stage, however, is a strong link between thoughts and action. During this stage, the level of egocentrism decreases, and the reversibility of operations is being shaped (Piaget, 1960; Harter, 1999). The period of middle childhood is a time of beginning school education. Therefore, younger children may have underdeveloped reading skills. The use of images facilitates the assimilation of information.

### Opportunities provided by picture-based assessment

The use of a traditional verbal questionnaire assumes that respondents are able to think abstractly about their personalities. Children aged 7–11 years (i.e., during middle childhood, or the stage of concrete operations in Piagetian tradition) have not yet developed these skills. Before 12 years of age, children are experience dependent, which means that their thinking processes are based on mental representations that relate to concrete events, objects, or experience (Case et al., 2001; Demetriou, 2004). This must be considered to adapt the measurement method to the level of cognitive development of the respondent as a child. Because abstract concepts are unintelligible for a child, it is worth trying to present an abstract concept based on concrete examples (which, for example, the child knows from daily life). It could increase the chance of correctly understanding of them. Very common abstract concepts—such as personality traits and values—are presented in the form of illustrations in children's books or movies (Harter and Pike, 1984). Additionally, using traditional questionnaires in studies on groups of children, it should be taken into account that children's reading skills may be limited.

Following this line of reasoning, it would seem reasonable to say that visual methods of personality measurement are more appropriate than verbal ones. Such visualizations represent specific situations, behavior, and the person to whom the child can easily relate. Furthermore, picture-based assessment, the concretization of incomprehensible abstract material, is more attractive than traditional questionnaires and helps interest the children and keep their attention (Harter and Pike, 1984; Schmalt, 2005). Similar reasoning was expressed, e.g., by Döring et al. (2010), when they developed the Picture-Based Value Survey for Children to measure values using Schwartz's (1992) approach, and by Valla et al. (1994, 2000) and Bergeron et al. (2013) when developing the Dominic Interactive to assess DSM-III-R-based diagnoses in children.

### A new measure—the pictorial personality traits questionnaire for children

To overcome the difficulties associated with measuring personality in children, we developed the Pictorial Personality Traits Questionnaire for Children (PPTQ-C), which was designed to consider the level of cognitive development of children as respondents. The main idea of this instrument is that the personality traits are indicated by pictures that represent behaviors. The character presented in each picture was designed to be unisex; therefore, the PPTQ-C is suitable for both boys and girls. Initially, the item pool comprised 25 items constructed in a deductive paradigm, and exemplar scenarios for each item were drawn by a professional graphic designer. Further, two competitive judges, personality psychologists, assessed the validity of the drawings by assigning each drawing to the corresponding scale that represented the five personality traits. Following the validation, a pilot study was conducted in a group of 219 children (44% girls) aged 9–13 years (*M*_age_ = 11.40; *SD*_age_ = 0.90). Analyses of the gathered data were conducted in Mplus (Muthén and Muthén, 1998–2012) and Jrule (Saris et al., 2009). Therefore, we not only based our analyses on global fit indices, but we also searched for misspecifications by considering the modification indexes and power of the test. As a result of those analyses, we eliminated the items that had the highest cross-loadings and correlated residuals (Maćkiewicz and Cieciuch, 2012).

The main goal of the current study was to establish the validity of the PPTQ-C among a large group of children. Our expectations regarding the PPTQ-C scales were as follows:

**Satisfactory factorial validity**. Because confirmatory factor analysis relies on an independent cluster model, it may not adequately represent reality as it is due to over restrictiveness (Marsh et al., 2010, 2014), which is present especially in Big Five research (Marsh et al., 2010). To test this hypothesis, we relied on exploratory structural equation modeling (ESEM; Asparouhov and Muthén, 2009; Morin et al., 2015). Because the response scale comprised only three options in the younger group of children and five options in the older group of children, we conducted our analyses on polychoric correlation matrices and chose the WLSMV as an estimation method with theta parameterization (Rhemtulla et al., 2012). To represent our theoretical assumption on the factor structure of the PPTQ-C, we used oblique target rotation and targeted each cross-loading to be close to zero; i.e., only items designed to measure extraversion were allowed to freely load on the extraversion factor, whereas items designed to measure other traits were assumed to load close to zero on the extraversion factor.
**Measurement invariance across gender**. Because personality traits are the most basic dispositional traits (McAdams, 1995), and the character presented in each item was designed as unisex, we hypothesized that the five factor structure will be invariant across boys and girls. This hypothesis was tested using multi-group ESEM with very same specification as described above.
**Satisfactory criterion validity**. This expectation was tested using the Multitrait-Multimethod Analysis (MTMM) procedure that was proposed by Campbell and Fiske (1959). In this procedure, one assumes that the correlations between two analogous scales, which are assessed by independent measures, will be higher than correlations between other scales.

## Methods

### Participants and procedure

First, the institutional board at the Psychology Institute, Cardinal Stefan Wyszyński University in Warsaw reviewed this project and gave us permission to implement it. The study was conducted in two groups: the first one comprised 501 children (51% girls) aged between 7 and 10 years old (*M*_age_ = 9.25; *SD*_age_ = 0.87) who were enrolled in 1st, 2nd, or 3rd grade in primary school; the second consisted of 527 children (50% girls) aged between 10 and 13 years old (*M*_age_ = 11.63; *SD*_age_ = 0.79) who were enrolled in 4th, 5th, or 6th grade in primary school.

The study was carried out in groups in the school classrooms. The study was anonymous and the children's participation required their parents' written consent. Those responsible for carrying out the study informed the children about the research and its aim. They also ensured that the respondents understood the instructions. If it was needed, the children were granted additional explanations on how to respond. Particular attention was paid to ensuring that children responded solely based on their own ideas and did not consult with each other.

### Instruments

#### Pictorial personality traits questionnaire for children

**Figure 1:**
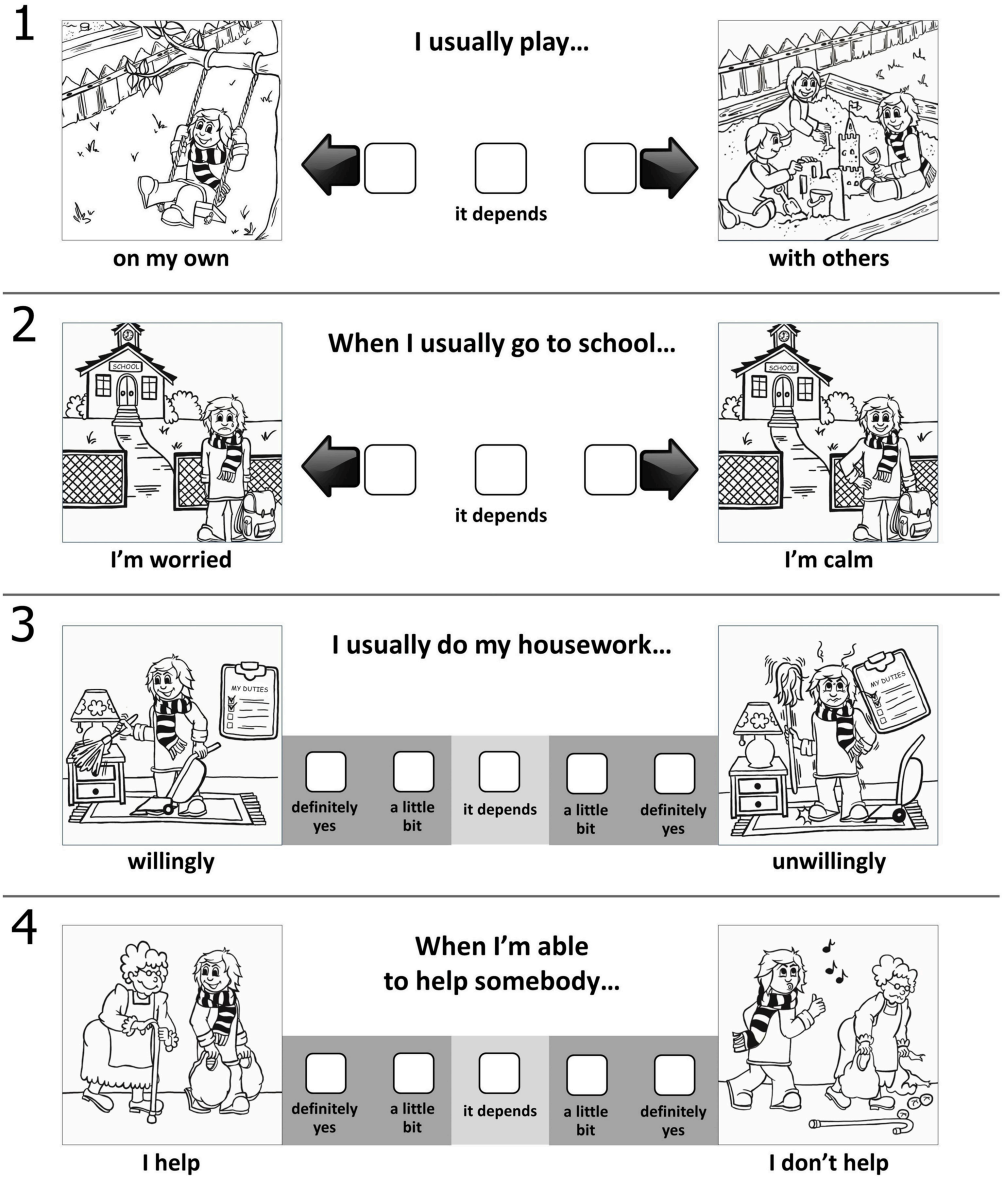
**The exemplary items of the PPTQ-C from version for younger and older children**. **1**, extraversion scale; **2**, neuroticism scale; **3**, conscientiousness scale; **4**, agreeableness scale.

The PPTQ-C consists of 15 items—three items for each scale: extraversion, neuroticism, openness to experience, agreeableness, and conscientiousness (see Supplementary Material). Each item consists of two pictures: the first is an indicator of a low level of a given trait, and the second indicates a high level of the same trait. The same main character is presented in each picture, although the character behaves in a different way. The child chooses the picture in which the main character behaves as he or she would and indicates his or her similarity to the main character on a 3-point (for younger children, 6–9 years old) or 5-point (for older children, 10–12 years old) response scale. Sample items are presented below in Figure 1.

The instructions explain that the child should first consider whether he or she more often behaves the same way as the main character in the left picture or the right one. Each child is then asked to select the box that indicates how often he or she behaves as shown in the picture. The response scale is highly similar to that created by Harter in the Self-Perception Profile for Children (Harter, 1985).

In the younger group of children, the reliability coefficients for the scales were as follows: extraversion α = 0.60, neuroticism α = 0.69, openness to experience α = 0.48, conscientiousness = 0.65, and agreeableness α = 0.69. In the older group, they were as follows: extraversion α = 0.50, neuroticism α = 0.62, openness to experience α = 0.44, conscientiousness = 0.61, and agreeableness α = 0.67. Reliability estimates for the younger group of children are higher; however, they are not high. It is worth noting that the estimate of Cronbach's α depends on the number of items in given scale (Sijtsma, 2009), and in PPTQ-C, there are only three items per scale. The lowest reliability estimates in both age groups were found for openness to experience. The standard deviations of each scale are listed in Table 1.

**Table 1:** **Means and standard deviations for the scales of PPTQ-C in both group of children**.

	**Younger children**		**Older children**	
	***M***	***SD***	***M***	***SD***
Extraversion	1.97	0.52	3.05	0.87
Neuroticism	1.50	0.67	2.39	1.01
Openness to experience	2.33	0.69	3.24	1.05
Conscientiousness	1.56	0.69	3.58	1.01
Agreeableness	2.35	0.61	3.08	0.87

#### Big five questionnaire for children

Moreover some of children aged 9 years and above completed the BFQ-C (Barbaranelli et al., 2003; Polish adaptation: Cieciuch et al., in press). The group of younger children was comprised of 142 children (52% girls) aged 9–10 years (*M*_age_ = 9.31; *SD*_age_ = 0.57). The group of older children was comprised of 193 children (51% girls) aged 10–13 years (*M*_age_ = 11.59; *SD*_age_ = 0.82).

The BFQ-C consists of 65 items (13 items for each of the five scales measuring the Big Five dimensions) with a 5-point response scale ranging from “almost never” to “almost always.” On this scale, children assess the frequency of their own behaviors by referring to the statements in each item. In the current study, the Cronbach's alpha reliability coefficients for the scales in the younger group were as follows: extraversion α = 0.84; neuroticism α = 0.88; agreeableness α = 0.91; conscientiousness α = 0.90; and openness α = 0.88. In the older group, the values were as follows: extraversion α = 0.84; neuroticism α = 0.87; agreeableness α = 0.89; conscientiousness α = 0.86; and openness α = 0.86.

## Results

### Factor structure of the PPTQ-C

**Figure 2:**
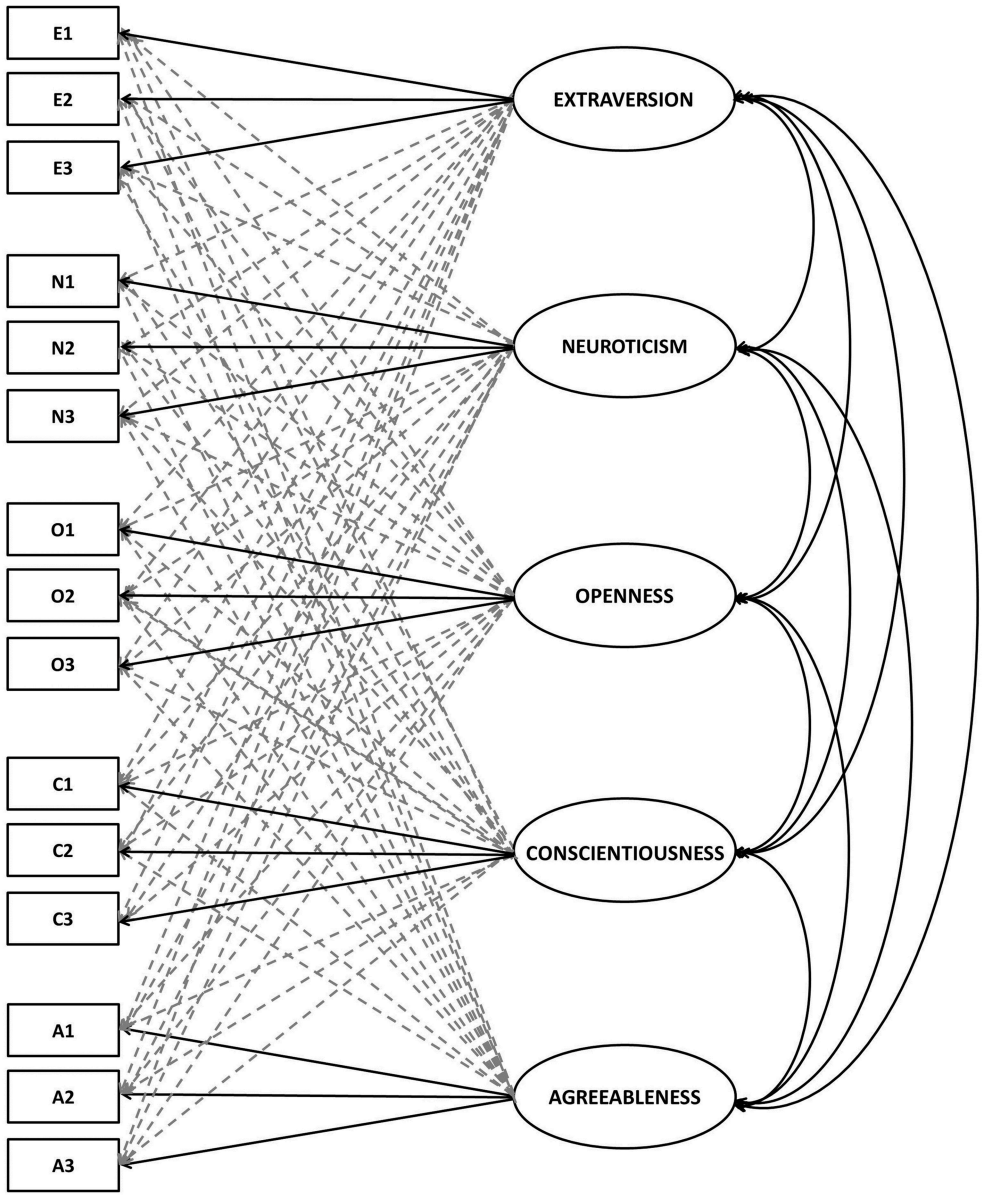
**Graphical representation of tested ESEM model**. Bolded lines represent expected factor loadings. Punctuated lines represent non-expected cross-loadings.

The analyses were carried out in Mplus v. 7.2 (Muthén and Muthén, 1998–2012). In assessment of model fit we relied on approximate fit indices (CFI, TLI > 0.95; RMSEA < 0.05; Hu and Bentler, 1999; Marsh et al., 2004), since χ^2^ is almost always significant when sample size is large (Kline, 2011). The conceptual graphical representation of tested ESEM models is presented in Figure 2.

The tested five-factor ESEM models were excellently fitted to the data in both the younger- [$\chi_{\left(40\right)}^{2}=$ 66.44; *p* = 0.005; CFI = 0.988; TLI = 0.969; RMSEA = 0.036 [90% CI = 0.020–0.051]; *p* = 0.931] and older-children groups [$\chi_{\left(40\right)}^{2}=$ 65.52; *p* = 0.007; CFI = 0.990; TLI = 0.973; RMSEA = 0.035 [90% CI = 0.018-0.050]; *p* = 0.955]. The standardized factor loadings of the models are presented in Table 2.

**Table 2:** **Standardized factor loadings of the five-factor ESEM model for younger- and older-children groups**.

	**Extraversion**	**Neuroticism**	**Openness to experience**	**Conscientiousness**	**Agreeableness**
**YOUNGER CHILDREN**					
1	**0.77**	0.12	−0.01	−0.04	0.00
6	**0.78**	−0.09	−0.01	−0.01	0.07
11	**0.48**	−0.13	0.17	0.10	−0.08
2	−0.03	**0.90**	0.03	0.10	−0.01
7	0.02	**0.46**	0.05	−0.34	−0.20
12	−0.17	**0.62**	−0.01	−0.10	0.13
3	0.01	0.04	**0.17**	0.24	0.30
8	0.00	0.01	**0.97**	−0.01	0.00
13	0.07	−0.04	**0.20**	0.45	0.14
4	0.06	0.08	−0.04	**0.82**	0.04
9	0.08	−0.05	0.05	**0.75**	−0.01
14	−0.18	−0.39	0.17	**0.35**	0.11
5	0.00	−0.09	−0.04	0.12	**0.63**
10	0.02	0.03	0.00	−0.04	**0.97**
15	0.03	−0.19	0.12	0.09	**0.55**
**OLDER CHILDREN**					
1	**0.63**	0.08	−0.03	−0.09	0.20
6	**0.64**	−0.12	−0.10	0.08	0.03
11	**0.20**	−0.18	−0.06	0.15	0.18
2	−0.08	**0.64**	−0.15	0.11	0.01
7	−0.02	**0.44**	−0.16	−0.26	−0.03
12	0.02	**0.87**	0.08	0.04	0.01
3	−0.09	0.04	**0.41**	0.16	0.23
8	−0.03	0.02	**0.14**	0.12	0.36
13	−0.10	−0.14	**0.47**	0.13	0.18
4	0.08	0.02	0.22	**0.64**	−0.09
9	0.08	0.00	0.10	**0.74**	−0.10
14	−0.19	−0.05	−0.32	**0.58**	0.26
5	0.06	−0.02	0.16	−0.14	**0.64**
10	0.07	−0.06	0.16	0.09	**0.56**
15	0.24	−0.07	−0.01	0.09	**0.54**

*The expected loadings are bolded, and non-expected cross-loadings are grayed*.

Most of the expected loadings were strong; however, there were a few exceptions. In the younger-children group, only one item from the openness to experience scale loaded as expected, and the remaining two items loaded this factor weakly. Therefore, the interpretation of this scale must be done with caution. Both of the items (3 and 13) from the openness to experience factors cross-loaded on the conscientiousness factor. Similarly, in the older-children group, we also found two weak factor loadings, one in the openness to experience factor and the second in the extraversion factor. The mean correlation between the latent factors was moderate in both groups (*Mr*_younger children_ = 0.32; *Mr*_older children_ = 0.30), and the highest correlation was found between agreeableness and conscientiousness (0.56 in younger children, and 0.44 in older children).

### Measurement invariance across boys and girls

Basically, there are three levels of invariance (configural, metric, and scalar; Meredith, 1993); however, because we treated our data as categorical, the metric and scalar measurement invariance level cannot be distinguished, and the equality of both loadings and intercepts are tested at one step (Millsap and Yun-Tein, 2004). In our assessment of measurement invariance, we relied on Chen's (2007) recommendations that a change between configural and metric levels of measurement invariance >0.01 in CFI, supplemented by a change of more than 0.015 in RMSEA, is an indicator of non-invariance. The same holds for the scalar measurement invariance compared to the metric level. Because those recommendations were developed for a continuous scale rather than an ordinal one, we interpreted them rather as a guide in our interpretation of model fit. Because we compared the scalar level to the configural level, we expected a change in CFI < 0.02 and a change in RMSEA < 0.03 (the sum of metric-configural and scalar-metric differences). The results of multi-group ESEM are presented in Table 3.

**Table 3:** **Model fit indices for five-factor multi-group ESEM model across gender for younger- and older-children groups**.

	**χ^2^_(df)_**	***p***	**CFI**	**RMSEA**
**YOUNGER CHILDREN**				
Configural	91.41_(80)_	0.180	0.995	0.024
Scalar	183.77_(140)_	0.008	0.980	0.035
Δ	–92.36	0.172	0.015	–0.011
**OLDER CHILDREN**				
Configural	112.54_(80)_	0.010	0.987	0.039
Scalar	211.87_(170)_	0.016	0.983	0.031
Δ	–99.33	–0.060	0.004	0.008

Both models were excellently fitted to the data at the configural and scalar levels, and the changes in CFI and RMSEA were less than the indicator of non-invariance. Thus, the results are gender invariant. Similarly, in the older-children group, ΔCFI and ΔRMSEA suggested that the results of boys and girls were invariant. Therefore, we compared latent mean scores across gender in both age groups using a *Z*-test, the results of which are presented in Table 4.

**Table 4:** **Gender differences across gender for younger- and older-children groups**.

	**Extraversion**	**Neuroticism**	**Openness to experience**	**Conscientiousness**	**Agreeableness**
Younger children	1.61	0.38	−0.28	1.32	2.01^*^
Older children	1.70	0.85	3.15^**^	1.03	2.55^*^

* *p < 0.05*.

** *p < 0.01*.

*Value greater than zero means higher level in girls*.

Girls scored significantly higher than boys in both age groups in agreeableness only. In the older-children group, girls also scored higher than boys in openness to experience. However, it is worth noting that this factor was comprised only by one item in the ESEM model; thus, the results for this scale should be interpreted with caution. In both groups, no differences were found for extraversion, neuroticism, and conscientiousness.

### Multitrait-multimethod analysis

To examine the criterion validity of the PPTQ-C we conducted a multitrait-multimethod analysis. Table 5 shows the correlations between traits measured by the PBPS-C and the BFQ-C in both the younger and the older children. In both groups, the correlations between the same traits measured by the PPTQ-C and the BFQ-C, as expected, were the highest among all of the correlations.

**Table 5:** **Multitrait-multimethod matrix for younger- and older-children groups**.

**PPTQ-C**							**BFQ-C**				
		**E**	**N**	**O**	**C**	**A**	**E**	**N**	**O**	**C**	**A**
PPTQ-C	E	–	−0.32^**^	0.18^**^	0.11^**^	0.40^**^	0.49 ^**^	−0.18^**^	0.26^**^	0.20^**^	0.52^**^
	N	−0.31^**^	–	−0.22^**^	−0.28^**^	−0.34^**^	−0.13	0.67^**^	−0.09	−0.12	−0.25^**^
	O	0.29^**^	−0.21^**^	–	0.38^**^	0.36^**^	0.30^**^	−0.02	0.67^**^	0.45^**^	0.38^**^
	C	0.24^**^	−0.39^**^	0.40^*^	–	0.25^**^	0.04	−0.19^**^	0.23^**^	0.51^**^	0.16^*^
	A	0.34^**^	−0.35^**^	0.38^**^	0.44^**^	–	0.40^**^	−0.22^**^	0.30^**^	0.24^**^	0.71^**^
BFQ-C	E	0.50^**^	−0.12	0.34^**^	0.33^**^	0.40^**^	–	0.28	0.48^**^	0.55^**^	0.45^**^
	N	−0.14	0.52^**^	−0.13	−0.19^*^	−0.11	0.08	–	0.16	0.03	−0.15^*^
	O	0.34^**^	−0.14	0.48^**^	0.44^**^	0.40^**^	0.72^**^	−0.03	–	0.71^**^	0.57^**^
	C	0.40^**^	−0.23^**^	0.40^**^	0.59^**^	0.40^**^	0.66^**^	−0.11	0.77^**^	–	0.54^**^
	A	0.40^**^	−0.21^*^	0.38^**^	0.42^**^	0.62^**^	0.70^**^	−0.14	0.65^**^	0.74^**^	–

*Correlations for the younger children's group (N = 142) are presented below the diagonal, and correlations for the older children's group (N = 198) are presented above the diagonal. E = extraversion, N = neuroticism, O = openness, C = conscientiousness and A = agreeableness*.

* p < 0.05;

** *p < 0.01*.

*The highest expected correlations were bolded*.

## Discussion

Despite the bold theses about the existence of the five universal factors of personality (McCrae, 2009) and the copious empirical evidence for the Big Five model's universality (McCrae, 2001; Hendriks et al., 2003; McCrae and Costa, 2003; McCrae et al., 2005), there appears to be a question about the developmental origins of these dimensions. An answer would be possible if developmental research used an instrument that was well adjusted to the developmental levels of the respondents. The goal of the current study was to provide a reliable and valid measurement of children's personality traits based on the Big Five model.

Using ESEM, we confirmed that the five factor structure of personality is distinguishable even in children aged 9 years. However, some developmental deviations were identified in both the younger and older groups of children. The most problematic scale was openness to experience. In younger children, the only item concerning intellectual curiosity loaded as expected by theory, whereas this very same item in older children had the weakest loading. A clue as to the potential explanation of this difference can be found within the cross-loadings onto the other factors. In the younger-children group, items that weakly loaded on openness to experience cross-loaded on the conscientiousness factor, whereas those in the older children's group that weakly loaded on openness cross-loaded on agreeableness factor. Younger children's activities are largely associated with school; thus, openness to experience and conscientiousness are both related to the importance of academic achievement (Herzhoff and Tackett, 2012). Thus, our results support the hypothesis derived from the informant approach to study children's personality (Mervielde et al., 1995; Mervielde and De Fruyt, 2000), i.e., that openness to experience is related with conscientiousness in children more than in adults. The second problem with the openness to experience scale is due its nature. Because openness to experience formally concerns very abstract ideas such as imagination, intellect, and sensitivity, it is either difficult or impossible to transfer those complex abstract ideas into a concrete, simple picture. Thus, one can conclude that the abstract character of openness to experience is reflected within the weakest results in this factor. The third and final problem with the openness to experience scale is the low reliability of the scale, particularly in the older-children group.

To summarize, we replicated the five factor structure in both age groups; however, the openness to experience scale is the one whose validity is problematic. Similarly to Lamb et al. (2002), we obtained reliability estimates close to 0.50, which is in conjunction with hypothesis that openness to experience is not well differentiated in childhood (Mervielde et al., 1995; Mervielde and De Fruyt, 2000). Contrary to that conclusion, Herzhoff and Tackett (2012) argued that openness to experience can be clearly identified in middle childhood; however, they used only observer reports. Thus, one can conclude that openness to experience could be identified using observer-reports rather than self-reports (Edmonds et al., 2013).

Similarly to Marsh et al. (2010), who applied the ESEM in the assessment of the Big Five personality structure in adults, we supported the thesis that the correlation strength between the factors found in other studies on children (Mervielde and De Fruyt, 2000; Barbaranelli et al., 2003) was jacked up by the overly restrictive confirmatory factor analysis. It is an unrealistic assumption that the Big Five factors are orthogonal (Marsh et al., 2010; Strus et al., 2014); thus, applying ESEM in personality research appears more appropriate (Marsh et al., 2010). Although, the mean correlation between latent factors was rather small in both age groups, it is worth noting that the correlation between agreeableness and conscientiousness was strongest. Soto et al. (2011) argued that children generally tend to accept the values and norms of adult authorities, which explains the high correlation between these two traits.

To date, the current study is the very first to report on the measurement invariance in young children across gender. We established scalar invariance; thus, we were able to compare the latent mean scores on each trait. Costa et al. (2001) and Weisberg et al. (2011), in large samples of adults, reported that women scored significantly higher than man in neuroticism, agreeableness, and extraversion (with the exception of assertive facets, where men scored higher) and in some facets of openness to experience, whereas no differences were found in conscientiousness. However, generally, those differences were rather small, and they were highest in agreeableness. Because in the current study, children differed only in agreeableness (we did not interpret the openness to experience in the group of older children due to methodological limitations), one can conclude that it is chronologically the first gender difference in personality. This finding is particularly important from an evolutionary perspective because agreeableness in women led to warmth, empathy, and emotional investments in others, which in turn enhanced the survivability of their children (Buss, 1995, 2008; Costa et al., 2001). Oppositely, men who were less agreeable were dominant, independent, and exploited others; thus, they gained an evolutionary advantage (Trivers, 1972; Schmitt et al., 2008; Weisberg et al., 2011). In the current study, we support the hypothesis that differences in agreeableness are rooted in evolution, and thus, it is chronologically the first gender difference in personality that can be observed.

To summarize, we demonstrated that PPTQ-C is a valid and reliable self-report measurement of personality traits. Also the results of the MTMM analysis were satisfactory in as much as they demonstrated convergence between the PPTQ-C and BFQ-C scales. Therefore, one can conclude that PPTQ-C is a valid measurement of personality traits in young children.

However, observer-report measures of openness to experience may be superior. It seems that a picture-based measurement of children's personalities can be an appropriate instrument for measuring children's personalities, particularly in a younger group of children (aged ~7–10 years). Thus, it fills the niche between the Berkeley Puppet Interview (Measelle et al., 2005), which is designed for children aged 5–7 years, and verbal questionnaires that can be used for older children (Barbaranelli et al., 2003). Future research may still focus on measurement invariance across gender in personality traits in older children to fill the gap in the literature between our study on childhood and studies (Costa et al., 2001; Weisberg et al., 2011) covering adulthood.

## Author contributions

MM: conception, data collection, analysis, and interpretation, writing the article. JC: statistical analysis, critical revision of the article.

## Funding

The work of MM was supported by Diamond Grant 0082/DIA/2012/41 from the Polish Ministry of Science and Higher Education. The work of JC was supported by Grants DEC-2011/01/D/HS6/04077 from the Polish National Science Centre.

### Conflict of interest statement

The authors declare that the research was conducted in the absence of any commercial or financial relationships that could be construed as a potential conflict of interest.
