# Supplementary material for: Pictorial Personality Traits Questionnaire for Children (PPTQ-C)—A New Measure of Children's Personality Traits
Source: Front Psychol. 2016 Apr 14;7:498. doi: 10.3389/fpsyg.2016.00498 (PMC4879772; doi:10.3389/fpsyg.2016.00498)
Supplement: Supplementary file 1 [file PPTQ-C_v1-questionnaire.pdf]

Jaki jesteś? Jak się zachowujesz?

OPCO v1, Marta Maćkiewicz i Jan Cieciuch

Na następnych stronach znajdują się opisy różnych sytuacji.
Oto przykładowa sytuacja:

„Kiedy pada deszcz…"

Sytuacje przedstawione są na obrazkach,
na których główną rolę odgrywa ta oto osoba w szaliku w paski,

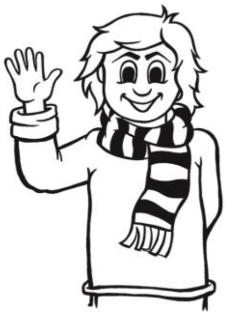

która na dwóch obrazkach robi dwie zupełnie różne rzeczy, np.

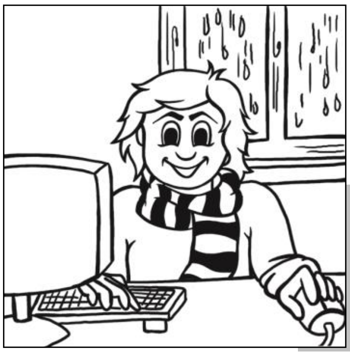

gram w gry na komputerze

Kiedy pada descz...

tak	trochę tak	różnie	trochę tak	tak
☐	☐	☐	☐	☐

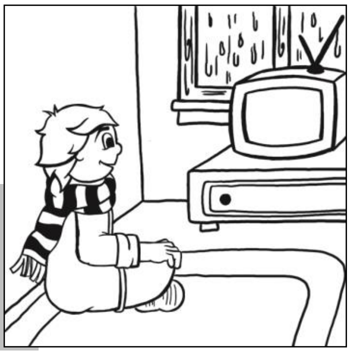

oglądam telewizję

Zastanów się, jak Ty **najczęściej** zachowujesz się w takiej sytuacji.
Czy jesteś bardziej podobny do osoby z szalikiem

na tym obrazku **czy** **na tym obrazku**

Kiedy już wybierzesz obrazek,
zastanów się, czy Twoje zachowanie jest
bardzo podobne („TAK"), czy tylko **trochę podobne („TROCHĘ TAK")**
do zachowania osoby z szalikiem z wybranego obrazka.
Następnie **zamaluj odpowiedni kwadracik** przy tym obrazku.

<u>Uwaga!</u>
W każdej sytuacji możesz wybrać **tylko jeden** kwadracik.

Jeśli czasem zachowujesz się tak, a czasem tak,
zamaluj środkowy kwadracik z podpisem „różnie".
Staraj się jednak **unikać** tego środkowego kwadraciku.
Za każdym razem zastanów się, **jak zachowujesz się częściej**.

Zanim zaczniesz, wpisz obok swoje imię, nazwisko, wiek oraz zaznacz podkreśleniem, czy jesteś chłopcem czy dziewczyną

imię i nazwisko...

wiek : ....

chłopiec / dziewczyna

A jak zachowujesz się Ty?

1. Zwykle bawię się…

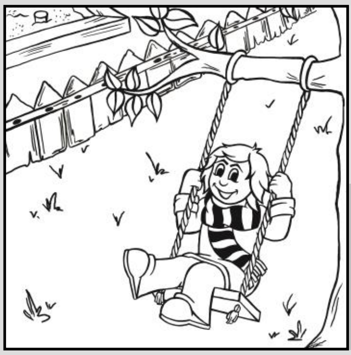

sam/sama

tak	trochę tak	różnie	trochę tak	tak
☐	☐	☐	☐	☐

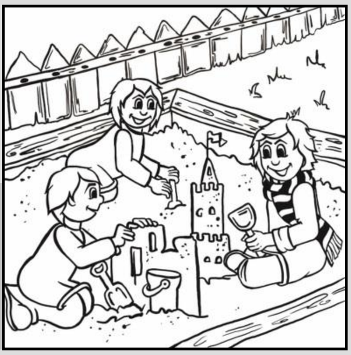

z innymi

2. Zwykle kiedy idę do szkoły…

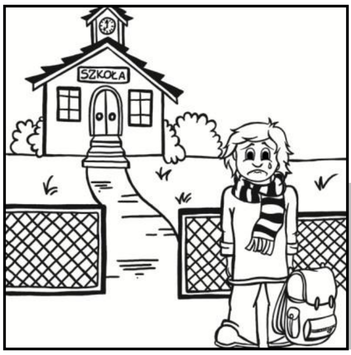

martwię się

tak	trochę tak	różnie	trochę tak	tak
☐	☐	☐	☐	☐

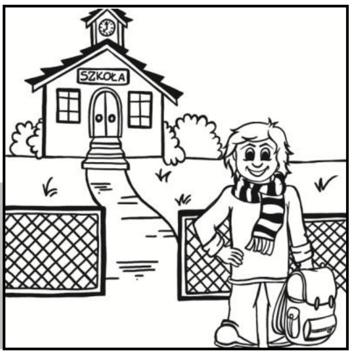

jestem spokojny/spokojna

3. Kiedy widzę lecące ptaki…

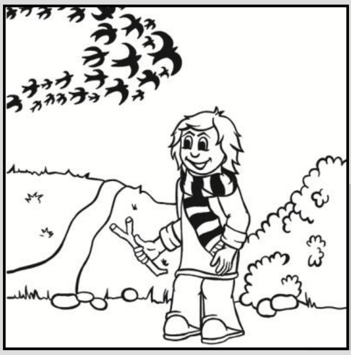

nie zauważam tego

tak	trochę tak	różnie	trochę tak	tak
☐	☐	☐	☐	☐

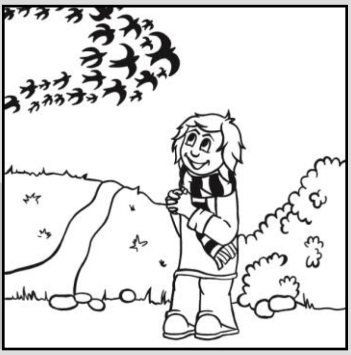

zauważam to

4. Domowe obowiązki wypełniam…

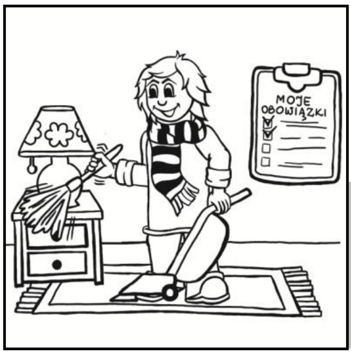

chętnie

tak	trochę tak	różnie	trochę tak	tak
☐	☐	☐	☐	☐

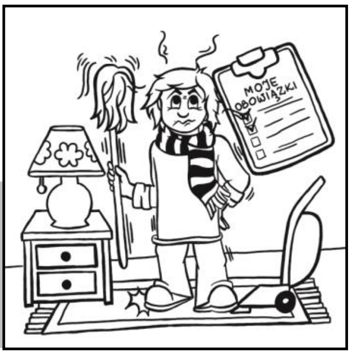

niechętnie

A jak zachowujesz się Ty?

5. Kiedy ktoś z mojej klasy czegoś potrzebuje...

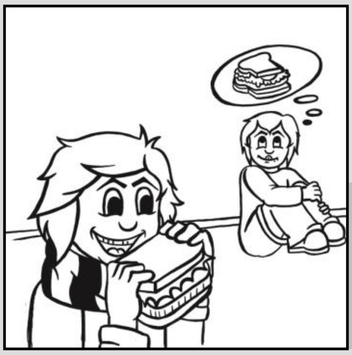

nie zauważam tego

tak	trochę tak	różnie	trochę tak	tak

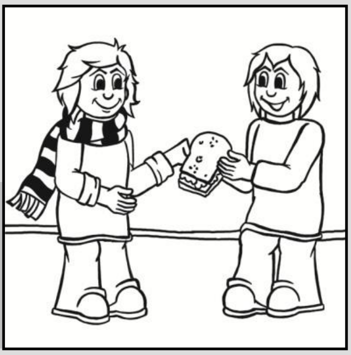

zauważam to

6. Kiedy inni bawią się...

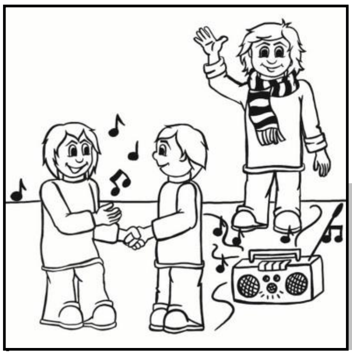

bawię się z nimi

tak	trochę tak	różnie	trochę tak	tak

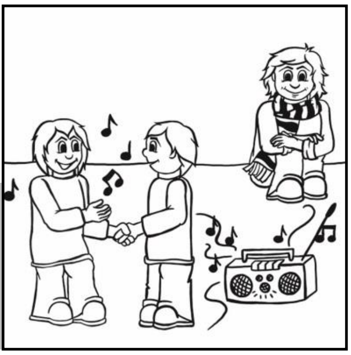

nie podchodzę do nich

7. Kiedy coś mi nie wychodzi...

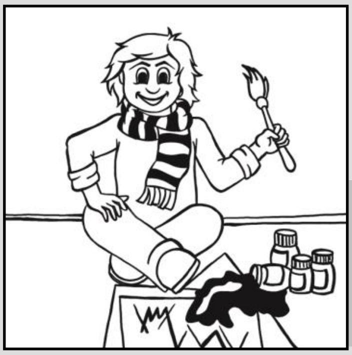

zachowuję spokój

tak	trochę tak	różnie	trochę tak	tak

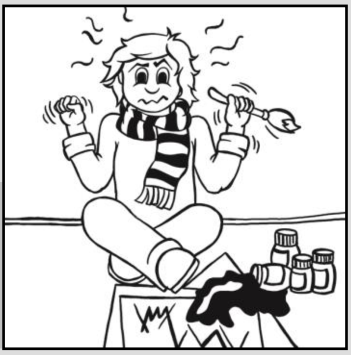

szybko się denerwuję

8. Na wycieczce lubię...

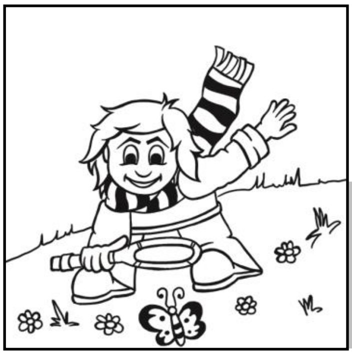

odkrywać coś nowego

tak	trochę tak	różnie	trochę tak	tak

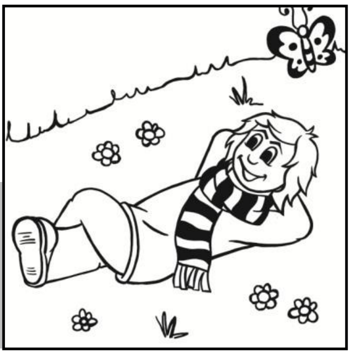

odpoczywać

A jak zachowujesz się Ty?

9. W moim pokoju...

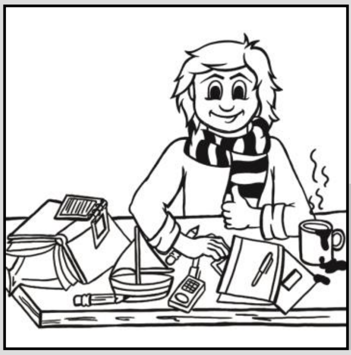

jest bałagan

☐	☐	☐	☐	☐
tak	trochę tak	różnie	trochę tak	tak

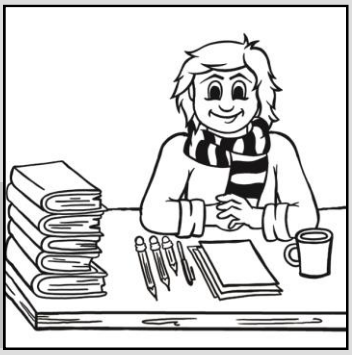

jest porządek

10. Kiedy widzę, że mogę komuś pomóc...

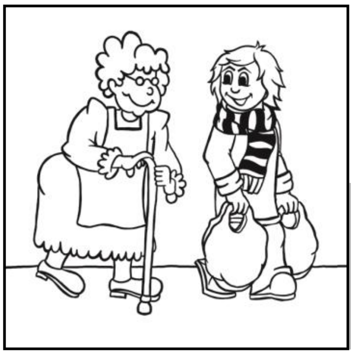

pomagam

☐	☐	☐	☐	☐
tak	trochę tak	różnie	trochę tak	tak

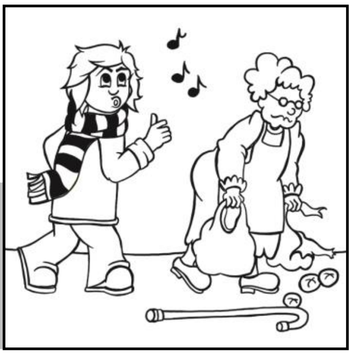

nie pomagam

11. Kiedy ktoś żartuje...

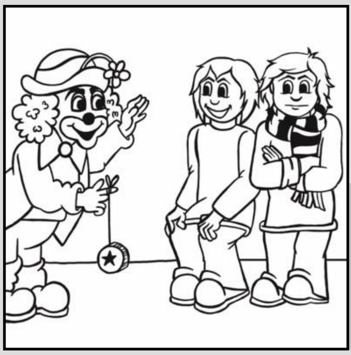

rzadko widzę w tym coś śmiesznego

☐	☐	☐	☐	☐
tak	trochę tak	różnie	trochę tak	tak

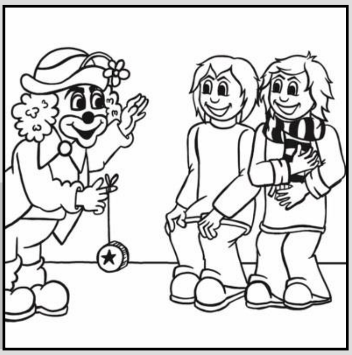

śmieję się z innymi

12. Zwykle...

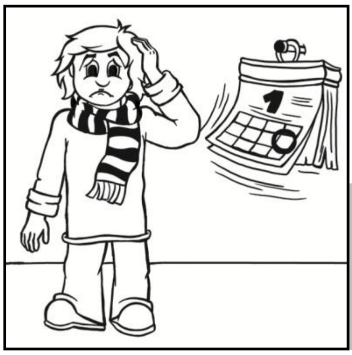

jestem spokojny

☐	☐	☐	☐	☐
tak	trochę tak	różnie	trochę tak	tak

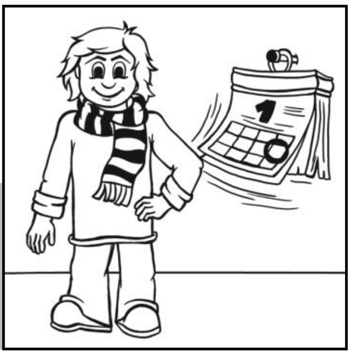

martwię się czymś

A jak zachowujesz się Ty?

13. Nowych i trudnych rzeczy…

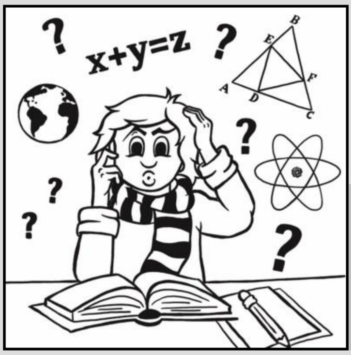

tak	trochę tak	różnie	trochę tak	tak
☐	☐	☐	☐	☐

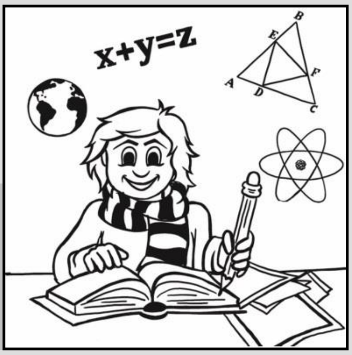

nie lubię się uczyć — lubię się uczyć

14. Kiedy dostanę od kogoś pieniądze…

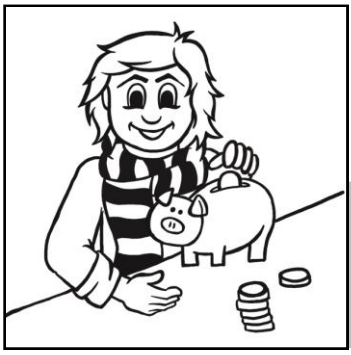

tak	trochę tak	różnie	trochę tak	tak
☐	☐	☐	☐	☐

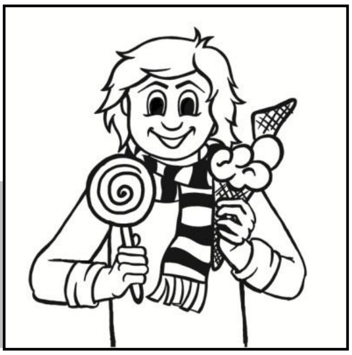

oszczędzam — wydaję je od razu

15. Kiedy mam nową zabawkę…

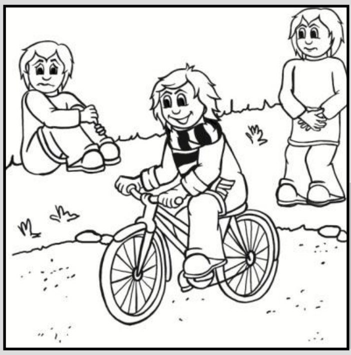

tak	trochę tak	różnie	trochę tak	tak
☐	☐	☐	☐	☐

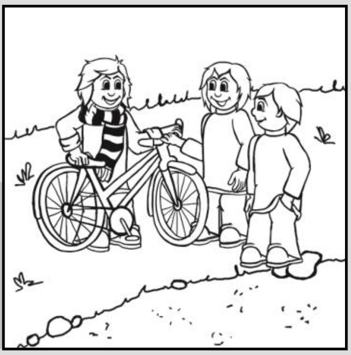

nie pożyczam jej innym — pożyczam ją innym

- **Extraversion:** 1, 6R, 11
- **Neuroticism:** 2R, 7, 12R
- **Openness:** 3, 8R, 13
- **Conscientiousness:** 4R, 9, 14R
- **Agreeableness:** 5, 10R, 15

Each item on the PPTQ-C (older children's version) is rated on a 5 point scale where:

- **Definitely** (left side)= 1 (5 for reverse scored items indicated by R)
- **A little bit** (left side) = 2 (4 for reserve scored items)
- **It depends** = 3
- **A little bit** (right side) = 4 (4 for reserve scored items)
- **Definitely** (right side side)= 5 (1 for reverse scored items)

Step 1: Reverse score the following items: 2, 4, 6, 10, 12, 14
Step 2: Compute scale scores by summing the items for each dimension
